# Pigmented Free-Floating Posterior Vitreous Cyst

**DOI:** 10.1155/2012/470289

**Published:** 2012-10-16

**Authors:** Claudia Bruè, Cesare Mariotti, Edoardo De Franco, Nicola De Franco, Alfonso Giovannini

**Affiliations:** ^1^Ophthalmology, Department of Neuroscience, Marche Polytechnic University, Ancona 60020, Italy; ^2^Department of Ophthalmology, I.N.R.C.A., Ancona 60127, Italy

## Abstract

Vitreous cysts are very rare ocular malformations. In this observational case study, we report on an unusual case of a pigmented free-floating vitreous cyst and discuss its differential diagnosis. A 14-year-old male was referred to ophthalmology for a pigmented lesion in his left eye. He complained of an intermittent floater in the left eye. Visual acuity was 20/20 in the right eye and 20/40 in the left eye. Fundus examination was unremarkable bilaterally, except for a piece of brownish oval material floating in the vitreous in the left eye. He had received a knock on the left side of his head a few days before the visual discomfort of the left eye. Real-time ultrasound of the left eye detected a piece of hyperechogenic spherical material with no internal reflectivity, floating in the middle of the vitreous. The first use of color Doppler ultrasound in this context indicated no arterial flow, ruling out the presence of a persistent hyaloid artery. Intraocular cysts are rare ocular disorders, which have been divided into clear and pigmented cysts, and into those that occupy the anterior chamber, the retrolental space, and the vitreous cavity. This last is extremely rare. We describe such a case.

## 1. Introduction

Vitreous cysts are particularly rare ocular malformations that can arise in an otherwise normal eye and also in diseased eyes or in association with the remnants of the hyaloid system [1]. The first description of a vitreous cyst was in 1899 by Tansley, as an irregularly spherical cyst that showed lines of pigment on its surface [2]. Several studies have discussed the possible origins of vitreous cysts, although no common agreement has been reached. We report here on the clinical and instrumental findings of an unusual pigmented free-floating cyst in the vitreous. 

## 2. The Case 

A 14-year-old male complained about a mobile shadow in his central visual field that he had had in his left eye for the preceding 3 months. He was born at 32 weeks of gestation. His ocular history was unremarkable. His medical history was positive for non-Hodgkin lymphoma, which had been cured following six courses of chemotherapy several years previously. He denied any ocular surgery.

The clinical assessment comprised a physical examination, blood tests, including a hemogram, serology for *Toxoplasma gondi*, *Toxocara*, Echinococcosis and cysticercosis, and a blood cell count for eosinophils. All of the blood tests were normal. Abdominal, thoracic, and brain magnetic resonance showed no pathological aspects. The patient referred to a history of head trauma during sports in the previous few years and a few days before the visual complaints. He did not report any inflammation or infections. 

Visual acuity was 20/20 in the right eye and had dropped from 20/20 to 20/40 in the left eye in the previous three months. When the floater did not interfere with the visual axis, visual acuity was also 20/20 in the left eye. Slit-lamp biomicroscopy revealed a normal anterior segment, with no signs of inflammation, and with iris translucency, transparent media, isochoric and photoreactive pupils, and a clear vitreous. His intraocular pressure was 14 mmHg bilaterally. 

**Figure 1:**
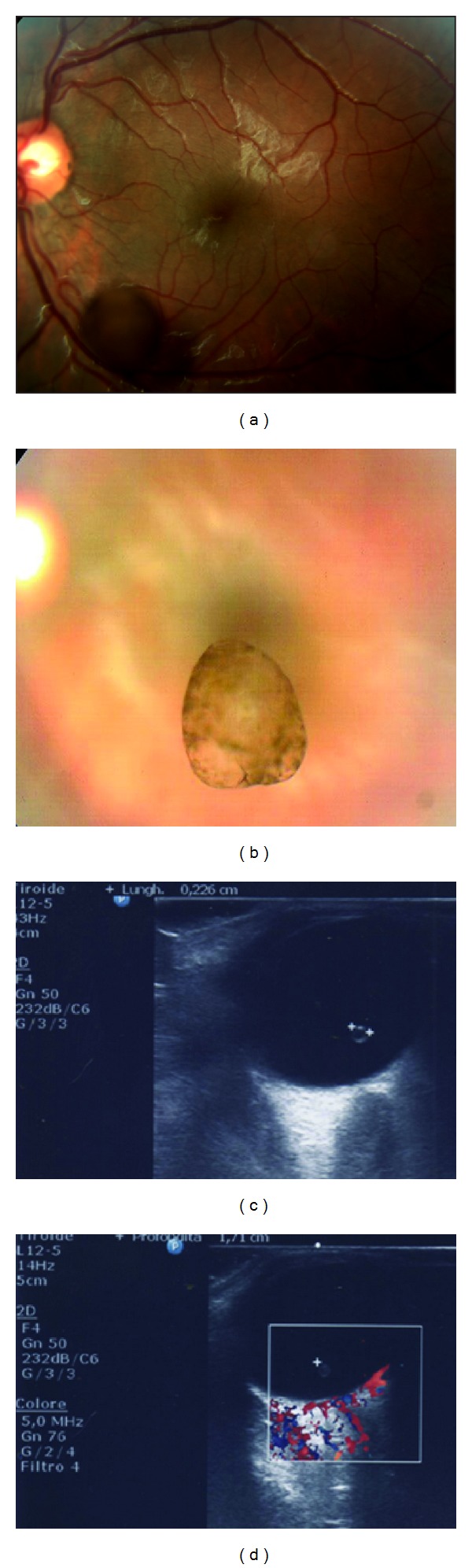
Ophthalmoscopy of the left eye shows a normal macula and a pigmented, oval-shaped cyst floating in the vitreous cavity and covering retinal vessels (a). The free-floating cyst was translucent, with a smooth brown-pigmented surface, and it was in the posterior vitreous (b). An oval-spherical hypoechogenic mass with thin hyper-reflective edges, with a 2.6-mm maximal diameter, was detected by real-time B-scan ultrasound of the left eye. The cyst was mobile in the posterior vitreous (c). Axial ultrasound with color Doppler showed no remnants of the hyaloid artery and no vessels in the vitreous of either of the globes, nor within the cyst (d).

Fundus examination of the right eye did not reveal any abnormalities. In the left eye there was a brownish, oval-shaped structure floating in the vitreous cavity, while the macula and peripheral retina appeared normal (Figure 1(a)). The cyst was translucent, with a smooth, brown-pigmented surface (Figure 1(b)). Gray-scale ultrasound evaluation (Gine Scan S, Quantel Medical, France) revealed a normal echogenic right eye. Real-time B-scan ultrasound of the left eye demonstrated an oval-spherical hypoechogenic mass with hyperreflective edges, with a 2.6-mm maximal diameter, which was floating at the posterior pole, and which was not attached to any other ocular structures (Figure 1(c)). No posterior vitreous detachment was detected. Color Doppler examination revealed an absence of vessels going through the vitreous of both globes and within the cyst (Figure 1(d)). No calcification was seen. Periodic observation was recommended without any treatment.

## 3. Discussion

Intraocular cysts have been classified into those that occur in the anterior chamber of the eye, those in the retrolental space, and those in the vitreous cavity [3]. Vitreous cysts are a sufficiently uncommon ocular disorder to be considered an “ocular curiosity.” This condition has been seen to occur in younger patients of 6–8 years old [4–6], across the whole range of adults, although it is seen mostly from 10 to 20 years of age. The numbers and positioning of such cysts include single monolateral, single bilateral, and multiple monolateral. Their dimensions can range from 0.15 mm to 12 mm, with shapes described as spherical, oval, and/or lobulated, while the surface can be smooth or sharp. Cysts can have a yellow-gray (nonpigmented) or brown (pigmented) appearance [1]. In symptomatic patients, the treatment options include laser photocystotomy or pars plana vitrectomy with cyst excision [7]. 

Although their origins are still debated, free-floating vitreous cysts have been classified mainly as congenital and acquired [1]. The former can originate from remnants of the hyaloid artery or glial remnants of Bergmeister's papilla, although they are occasionally detected in normal eyes [1, 8]. These congenital vitreous cysts have been depicted as sessile or pedunculated pearl-gray cysts that are located anterior to the optic disc, and rarely they are limited in their movements by vitreous strands that are linked to the optic disc. Acquired vitreous cysts have been described in patients affected by degenerative diseases, such as retinitis pigmentosa [1] and choroidal atrophy [1], and have been reported to arise from degeneration of a ciliary adenoma breaking into the vitreous cavity [3], cystic growth at a site of a coloboma that enters the vitreous cavity [3], uveitis [1], toxoplasmosis [1], retinal detachment [1], retinoschisis [1], parasitic vitritis, and nematode endophthalmitis [3]. 

Our patient denied intravitreal injections, travel abroad, or systemic inflammation during the past few years. Parasitic cysts usually have thick walls and are cream-white in color [9]. Furthermore, the absence of vitritis, retinitis, retinal and optic-nerve perivasculitis with extensive degeneration of the peripheral retina, and pigmentary retinal tracks, taken in conjunction with a normal blood test, excluded nematode or parasite infection. 

Orellana et al. analyzed the vitreous cysts of two patients, both with a positive history of trauma. Using electron microscopy, they detected mainly large mature melanosomes associated with scatter immature melanosomes, which led to their assumption that these cysts were secondary to the trauma and were generated from the pigment epithelium [3]. The pigment can arise from the pigmented ciliary epithelium, as probably the pars plana ciliaris, which can be separated by trauma and migrate into the vitreous, forming a pigmented cyst. Awan came to a similar conclusion after the detection of vitreous cysts in 2.7% of patients with a history of trauma [10]. 

However, other studies have argued the significance of trauma in the development of vitreous cysts, proposing that trauma might dislocate a preexisting vitreous cyst along the visual axis [1]. This is supported by the appearance of congenital cysts, which are almost always translucent, including those that are covered by pigment epithelial cells, such as traumatic cysts. On the other hand, acquired cysts usually appear as opaque or only slightly translucent [9]. Nork et al. carried out histopathological examination of a vitreous cyst, where they described pigment-epithelial-type tissue with immature melanosomes. As this was in the region of Cloquet's canal, where there was an anterior remnant of the hyaloid artery, known as a Mittendorf's dot; this reinforced the hypothesis of the origin being the primary hyaloidal system.

The incomplete regression of the hyaloidal vasculature can be investigated in terms of the presence of blood flow using color Doppler ultrasound [11]. To the best of our knowledge, our study represents the first use of color Doppler ultrasound in this context of a pigmented free-floating vitreous cyst. This color Doppler ultrasound with our patient did not show any arterial flow, both in terms of an absence of vessels going through the vitreous of both globes and within this vitreous cyst, ruling out the presence of a persistent hyaloid artery. 

As our case presented with a history of trauma, and showed ultrasonographic and clinical findings similar to those described by Orellana et al. [3], this led to the diagnosis of a free-floating vitreous cyst due to trauma. However, cases of pigmented free-floating vitreous cysts are extremely rare, so these lesions are of note. The differentiation of the congenital and acquired forms is meaningful, to establish the correct management, and further studies are needed to fully understand the pathogenesis and the course of this disease.
